# *Ac*/Ds-transposon activation tagging in poplar: a powerful tool for gene discovery

**DOI:** 10.1186/1471-2164-13-61

**Published:** 2012-02-06

**Authors:** Matthias Fladung, Olaf Polak

**Affiliations:** 1 Johann Heinrich von Thuenen-Institute Federal Research Institute for Rural Areas, Forestry and Fisheries Institute of Forest Genetics Sieker Landstr. 2 D-22927 Grosshansdorf Germany

**Keywords:** functional genomics, Populus, mutant, tree genomics, transgenic aspen, transposition

## Abstract

**Background:**

Rapid improvements in the development of new sequencing technologies have led to the availability of genome sequences of more than 300 organisms today. Thanks to bioinformatic analyses, prediction of gene models and protein-coding transcripts has become feasible. Various reverse and forward genetics strategies have been followed to determine the functions of these gene models and regulatory sequences. Using T-DNA or transposons as tags, significant progress has been made by using "Knock-in" approaches ("gain-of-function" or "activation tagging") in different plant species but not in perennial plants species, e.g. long-lived trees. Here, large scale gene tagging resources are still lacking.

**Results:**

We describe the first application of an inducible transposon-based activation tagging system for a perennial plant species, as example a poplar hybrid (*P. tremula *L. *× P. tremuloides *Michx.). Four activation-tagged populations comprising a total of 12,083 individuals derived from 23 independent "Activation Tagging Ds" (ATDs) transgenic lines were produced and phenotyped. To date, 29 putative variants have been isolated and new ATDs genomic positions were successfully determined for 24 of those. Sequences obtained were blasted against the publicly available genome sequence of *P. trichocarpa *v2.0 (Phytozome v7.0; http://www.phytozome.net/poplar) revealing possible transcripts for 17 variants.

In a second approach, 300 randomly selected individuals without any obvious phenotypic alterations were screened for ATDs excision. For one third of those transposition of ATDs was confirmed and in about 5% of these cases genes were tagged.

**Conclusions:**

The novel strategy of first genotyping and then phenotyping a tagging population as proposed here is, in particular, applicable for long-lived, difficult to transform plant species. We could demonstrate the power of the ATDs transposon approach and the simplicity to induce ATDs transposition *in vitro*. Since a transposon is able to pass chromosomal boundaries, only very few primary transposon-carrying transgenic lines are required for the establishment of large transposon tagging populations. In contrast to T-DNA-based activation tagging, which is plagued by a lack of transformation efficiency and its time consuming nature, this for the first time, makes it feasible one day to tag (similarly to Arabidopsis) every gene within a perennial plant genome.

## Background

One of the global challenges for the next decades is the reproducible and sustainable production of wood to meet the increasing demand for energy and solid raw material. The majority of the terrestrial biomass is produced by forest trees, which are grown either in natural (primeval and secondary) forests or, with increasing significance, in tree plantations. Plantation forestry is predicted to become even more important in the future to reduce the pressure on primeval forests in an effort to support ecologically sustainable and economically profitable wood production. One substantial opportunity for plantation forestry lies in the ability to use improved domesticated tree varieties or even genetically modified (GM) trees, specifically designed for a respective end-use, e.g. low-lignin trees for pulp and paper or saccharification (bioethanol production), or high-lignin trees for solid wood combustion.

Improving trees by conventional breeding is time-consuming and often not cost-effective due to the long vegetative periods and long reproduction cycles [1]. The availability of whole genome sequences of forest trees offers the opportunity to detect novel genes responsible for important developmental processes like tree growth or wood production. In combination with the publicly accessible whole genome sequences for *Populus trichocarpa *[2] and *Eucalyptus grandis *(http://eucalyptusdb.bi.up.ac.za/), the development of new genomic tools like "Target Induced Local Lesions IN Genomes" (TILLING, [3]) or the production of genotypes carrying novel (desired) gene combinations offer the opportunity to fasten tree domestication.

The *P. trichocarpa *genome is approximately 403 Mb in size, arranged in 19 chromosomes and assembled into 2,518 scaffolds. The number of loci containing protein-coding transcripts is 40,668, but 45,033 protein-coding transcripts have been detected (annotation v2.2 of assembly v2.0; Phytozome v7.0; http://www.phytozome.net/poplar). However, only for a minority of these loci the functions of the protein-coding transcripts are positively known. For tree species including poplar, only very few mutants have been described that could be used to analyse specific gene function behind the mutation [4]. Induced mutagenesis combined with phenotyping tools offer significant opportunities for linking gene models with putative functions. Over the past decade, genomics reagents have become available to produce a wealth of tagged mutant plants in particular for annual model species. Mutant induction in such annual plants by T-DNA insertion or using the mobility of transposable elements (e.g. the maize *Ac *element or its inactive derivate Ds) in most cases was achieved using knock-out tagging, disrupting a functional pathway by element insertion in functional genes and subsequent selfing of mutagenized plants. In *Arabidopsis*, it is now possible to acquire a mutant of nearly every gene model by using publicly available populations of T-DNA [5,6] or transposon [7,8] insertional mutagenesis lines. Similarly, large scale gene tagging resources have been developed for rice [9,10]) and barley [11,12].

The use of loss-of-function mutations described above is not well suited for application in long-living trees. In contrast, gain-of-function strategies have significant advantages because affected genes are not disrupted but activated [13-15]. One gain-of-function approach is "Activation tagging" which means the up-regulation of an endogenous gene through presence of a tag containing strong enhancers [16] or promoters facing outwards [17,18]. The concept behind transformation-based activation tagging is that the enhancers or the promoter are located on the T-DNA (or the transposon), and following insertion of the T-DNA close to a gene, its transcription will be activated. For Arabidopsis, large sets of "activation tagging populations" have been generated containing several T-DNA-based activation tagging vectors which are readily available from insertion collections and stock centers [19,20].

Despite the publication of some promising reports that describe the creation of T-DNA-based activation-tagged populations in poplar [15,21] and the identification of *GA2-OXIDASE*, a dominant gibberellin catabolism gene, as the first gene to be isolated from such a population [22], efficient gene tagging system for long lived forest tree species are still wanting. In order to fill this gap, Fladung et al [13] and Kumar and Fladung [23] proposed the use of a transposon-based activation tagging system for poplar. This proposal was based on the fact that the maize transposable element *Ac *is functional in the *Populus *genome [24], and re-integrations occur at high frequencies in or near coding regions [23]. Further, the majority of re-integrations were found scattered over many unlinked sites on other scaffolds than the one carrying the original integration locus, confirming that *Ac *does in fact cross chromosome boundaries in poplar [25].

In this paper, we describe for the first time the development of an efficient activation tagging system for aspen-*Populus *based on a non-autonomous "Activation Tagging Ds" (ATDs) system as described by Suzuki et al [26], in combination with a heat-inducible *Ac*-transposase. Four activation-tagged populations comprising in total 12,083 individuals have been produced and phenotyped. Many of the phenotypes have not been described before. Molecular analyses of individuals of the mutant population confirm the excision of the ATDs element from the original insertion locus and re-integration into or close to a gene locus, with unknown function in many cases. In a second, "blind" approach (without any phenotypic selection), 300 randomly selected individuals were PCR-screened for ATDs excision. In approximately one third of the investigated individuals, ATDs transposition was confirmed and analyses of the new genomic positions of ATDs reveal a very high percentage of tagged genes.

This system might prove particularly useful not only in poplar but also in other long-lived forest and fruit tree species where T-DNA-based activation tagging systems are not reliable due to the lack of high-efficiency transformation protocols.

## Results

### Production of transgenic plants and molecular analysis

From the seven independent HSP::*TRANSPOSASE *transgenic lines obtained, two transgenic lines, N66-2 and N66-5, were selected for super-transformation with p7N-ATDs-*rolC *guided by the results of PCR (presence of construct) and RT-PCR experiments (highest transposase transcript abundance; data not shown). Both lines were shown to carry one copy of the HSP::*TRANSPOSASE *gene (Table 1). The genomic insertion loci were identified on scaffold 3 at positions 16,990,223 and 15,414,366 for line N66-2 and N66-5, respectively (Table 1). Both insertion loci sequences showed high similarities to *P. trichocarpa *transcripts, for N66-2 to POPTR_0003s17690 with no functional annotation, and for N66-5 to POPTR_0003s15650 with functional annotation to CTP synthase (UTP-ammonia lyase) (Table 1).

**Table 1:** Copy number of HSP::*TRANSPOSASE *in the single transgenic lines, and genomic insertion locus (scaffold and position) with score, e-value and, if applicable, annotated transcript.

Transgenic line	Copy number	Genomic insertion locus (scaffold:position)*	Score	E- value	Transcript
N66-2	1	3:16,990,223	1132.0	0	POPTR_0003s17690 (no functional annotation)

N66-5	1	3:15,414,346	1222.2	0	POPTR_0003s15650 CTP synthase (UTP-ammonia lyase)

*based on BLAST-results against the genome sequence of *P. trichocarpa *v2.0 (Phytozome v7.0; http://www.phytozome.net/poplar). Successful positioning of blasted sequence on the physical map of *P. trichocarpa *was assigned to the *Populus*-aspen genome because of the high collinearity between the *P. trichocarpa *and *P. tremula/P. tremuloides *genomes [49].

**Figure 1:**
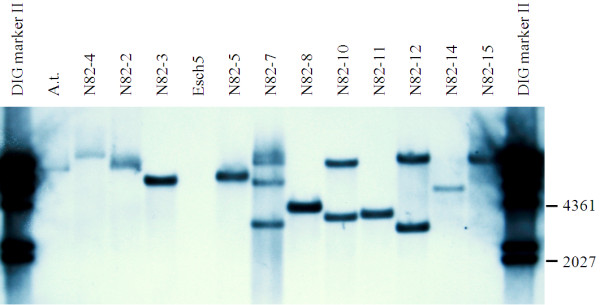
**Southern blot analysis of transformed poplar lines carrying the plasmids p6-HSP-TP-OCS and p7N-ATDs-*rolC***. A representative blot with *Sca*I restricted and *nptII*-probed DNA isolated from *Agrobacterium *strain used for transformation, negative control line Esch5, and eleven transgenic lines N82-2 to -5, N82-7 to -8, N82-10 to -12, N82-14 to -15 is shown. *Sca*I has only one restriction site in the cassette that can be used for copy number determination. Genomic DNA was separated on agarose gel, blotted and hybridized with a DIG-labelled *nptII *probe. A.t.: positive control (*Agrobacterium tumefaciens*), Esch5: non-transformed control line.

Super-transformation of N66-2 and N66-5 with p7N-ATDs-*rolC *yielded 23 double transgenic lines (twelve for N66-2 and eleven for N66-5) carrying the ATDs-*rolC *gene construct (data not shown). Using Southern blot analyses, the copy number of the ATDs-*rolC *gene could be determined for 21 double transgenic lines: 16 carried one copy, 4 lines two copies, and 1 line four copies (Table 2). Figure 1 shows a representative Southern blot with *Sca*I restricted and *nptII*-probed DNA isolated from eleven transgenic lines from the N82 group.

**Table 2:** Copy number of ATDs-*rolC *in the double transgenic lines, and genomic insertion locus (scaffold and position) with score, e-value and, if applicable, annotated transcript.

Transgenic line	Copy number	Genomic insertion locus (scaffold:position)*	Score	E-value	Transcript (POPTR_)
N82-2	1	18:5,952,389	841.7	0	---

N82-3	1	7:3,347,471	854.3	0	0007s05190 no functional annotation

N82-4	1	8:2,414,980	580.2	5.0e-164	0008s04320 Cyclin, N-terminal domain

N82-5	1	607:11,508	132.9	1.4e-29	0607s00230 no functional annotation

		18:4,994,998	165.4	1.7e-39	0018s05440 Ankyrin Repeat-Containing
N82-7	4	7:9,643,105	71.6	2.5e-11	**---**
		**2:14,852,606**	**41.0**	**2.2e-2**	**---**
		n.d.**			

N82-8	1	18:1,568,406	1463.8	0	POPTR_0018s01710
		or			F-Box/Leucine Rich Repeat Protein
		3:13,577,759	1551.2	0	POPTR_0003s13180 Heat Shock Protein 90

N82-10	2	2:10,740,018	612.2	5.3e-174	0002s14460 Zinc-finger double stranded
		5:9,290,567	91.5	3.4e-17	0005s12520 GRAS family transcription factor

N82-11	1	16:13,606,858	879.5	0	POPTR_0016s14360 Elongation factor P, C-terminal

**N82-12**	**2**	**17:7,632,882**	**44.6**	**9.8e-3**	**---**
		n.d.**			**---**

N82-14	2	3:13,408,914	322.3	1.9e-89	---
		n.d.**			---

N82-15	1	2:21,183,733	199.7	6.8e-50	---

N92-1	1	15:6,453,477	286.2	1.6e-75	---

N92-2	1	2:10,740,357	706.4	0	---

**N92-3**	**1**	**11:16,469,472**	**39.2**	**2.0e-3**	**POPTR_0011s13850 (COBRA-like protein)**

N92-4	1	10:15,737,708	515.2	2.1e-144	---

N92-5	1	10:19,481,904	417.9	4.4e-115	---

N92-6	n.d.**	n.d.**			

N95-1	1	n.d.**			

N95-2	2	25:155,725	1490.9	0	POPTR_0025s00400 (CYTH domain)
		**15:824,499**	**48.2**	**4.2e-4**	**---**

N95-3	1	9:9,143,423	291.6	3.0e-77	POPTR_0009s10720 (Arylacetamide deacetylase)

**N95-4**	**1**	**17:7,632,882**	**44.6**	**3.2e-3**	**---**

N95-5	1	16:7,726,062	562.1	1.4e-158	---

N95-6	n.d.**	n.d.**			

In bold: blast-results with high e-values. In BLAST-analyses where more than one hit was given, either the one with lower e-value or when similar, both hits are shown.

*based on BLAST-results against the genome sequence of *P. trichocarpa *v2.0 (Phytozome v7.0; http://www.phytozome.net/poplar). Successful positioning of blasted sequence on the physical map of *P. trichocarpa *was assigned to the *Populus*-aspen genome because of the high collinearity between the *P. trichocarpa *and *P. tremula/P.tremuloides *genomes [49].

**n.d. = not determined.

In 20 double transgenic lines, genomic sequences flanking the insertion locus of the second T-DNA could be successfully located on 13 different scaffolds, although in 3 one-copy lines and in 2 two-copy lines e-values were only marginal (bold in Table 2). For BLAST-analyses that resulted in more than one hit, either the hit with lower e-value was considered, or when similar e-values were obtained, both hits are shown in Table 2. Three of four ATDs copies from line N82-7 could be positioned in the genome, one with low, one with intermediate and one with a high e-value (Table 2). Genomic sequences from ten of the 20 lines showing successful T-DNA insertion allowed positive transcript annotation (Table 2).

All aspen-specific sequences obtained in this study were integrated into GabiPD database (http://www.gabipd.org) and submitted to GenBank ([GenBank:JM973488] to [GenBank:JM973566]).

### Heat shock experiments and ATDs excision

**Figure 2:**
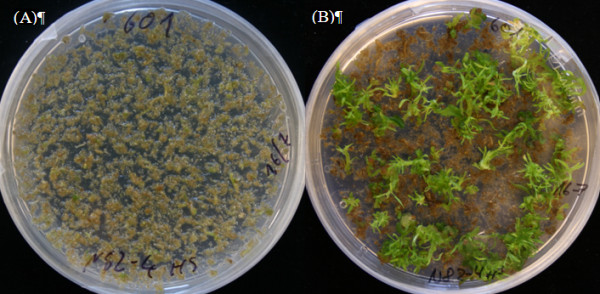
**Following heat shock treatment, regenerative callus, leaves and stems were crushed into pieces as small as possible in a Waring blender**. (A) The resulting "cell-pulp" was transferred to petridishes containing fresh regeneration medium and cultivated for up to 5 months at 25°C and continuous light in the growth chamber. (B) Regenerated shoots.

To induce ATDs transposition, four different heat shock experiments were conducted using a total of 23 independent double transgenic HSP::*TRANSPOSASE*/ATDs aspen lines (Table 3). Following the heat shock, plant material was crushed into pieces as small as possible and transferred to hormone-containing medium to regenerate shoots (Figure 2). Successfully regenerated shoots were cut, transferred to WPM medium without hormones for rooting, and rooted plants were phenotyped in tissue culture or in soil after three to six months growth in the greenhouse.

**Table 3:** Heat shock treatment experiment, treatment conditions, transgenic lines treated and number of in vitro rooted plants cultivated in greenhouse

Heat shock experiment	Treatment conditions	Transgenic lines treated	Rooted plants phenotyped in greenhouse
1	42°C for 16 to 24 hours	N82-2, N82-3, N82-4, N82-5, N82-7, N82-10, N82-11, N82-12, N82-14, N82-15	7,856

2	42°C for 24 hours	Esch 5 (control), N 92-1, N 92-2, N 92-3, N 92-4, N 95-1, N 95-2, N 95-3, N 95-4, N 95-5, N 95-6	623

3	Three days at 42°C for 8 hours, recovering over night	N82-2, N82-5, N82-7, N82-11, N82-14, N82-15	1,587

4	42°C for 24 hours	N 92-1, N 92-2, N 92-3, N 92-4, N 92-5, N 92-6, N95-1, N 95-2, N 95-3, N 95-4, N 95-5, N 95-6	2,017

			12,083

To confirm that the PCR fragment generated with the primer pair 16/37 contains the ATDs empty donor site, PCR fragments from 18 plants deemed to be positive for ATDs excision were sequenced. All sequences revealed the typical -GCCG- or -GGCG- linkage sequence between the *npt-II*-T35S and the *rolC *fragments, thus clearly indicating ATDs excision (data not shown).

### Phenotyping in four tagging populations

**Figure 3:**
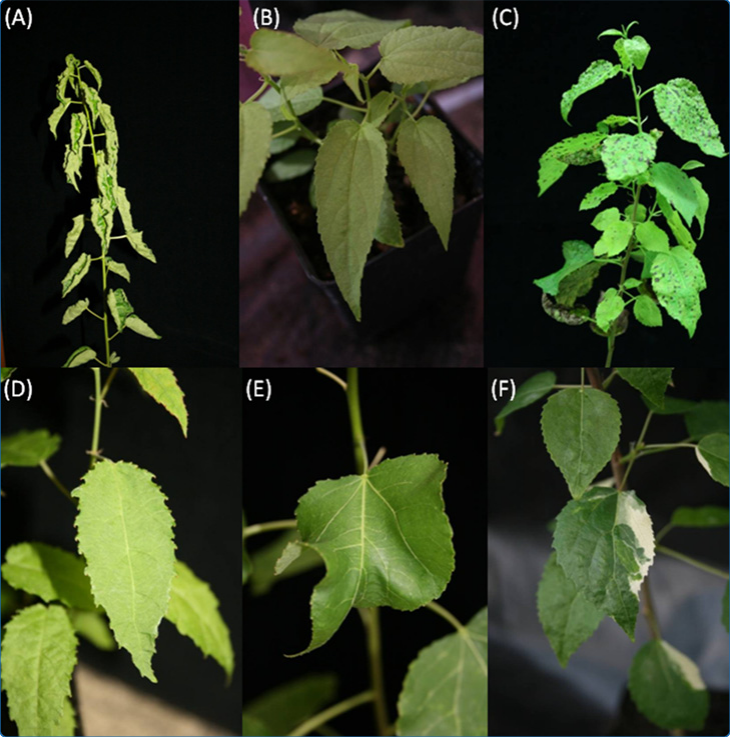
**Examples of pronounced phenotypes of ATDs tagged poplar lines**. (A) N82-3-66 (shriveled leaf), (B) N82-5-3 (lanceolated leaf), (C) N82-5-20 (necrotic leaf), (D) N82-11-1 (crippled growth in vitro, saw toothed leaf), (E) N82-14-10 (undulating leaf), (F) N92-1-6 (variegated leaf).

In total, 12,083 plants from 23 different ATDs transgenic lines were screened for phenotypic variation, mainly growth deficiency, chlorophyll abnormalities, and alterations in leaf form and shape. Twenty nine different phenotypic variants were detected, most of them remaining stable at least 12 month in tissue culture and/or in the greenhouse, as well as in copies generated by cuttings. Some phenotypes disappeared following the first winter period (data not shown) even if the ATDs insertion locus remained unchanged. A summary of detected phenotypes as well BLAST- and annotation results of new ATDs flanking sequences is presented in Table 4. Examples of pronounced phenotypes are shown in Figure 3.

**Table 4:** Pronounced phenotypes of ATDs tagged poplar lines: annotation results of new ATDs flanking sequences.

Transgenic line	Number of variants	Variant affiliation	Phenotype	Genomic insertion locus (scaffold:position)*	Score	E-value	Transcript (POPTR_)	Functional annotation
		N82-2-11	Variegated leaf	n.d.*				
		
		N82-2-13	Variegated leaf	18:5,952,981	154.6	1.5e-35		
		
N82-2	5	N82-2-14	Dwarf plant, weakly shriveled leaf	18:5,946,821	253.8	3.6e-66	0018s06220	2OG-Fe(II) oxygenase superfamily
		
		N82-2-64	Shriveled leaf	1599:3,445	250.2	3.8e-65		
		
		N82-2-70	Wavy leaf	3:15,139,843	702.8	0	0003s15320	Hypothetical protein (basic region leucine zipper)

		N82-3-23	Serrated leaf	10:20,130,280	206.9	5.7e-52	0010s23600	1,4-alpha-glucan branching enzyme/starch branching enzyme II
		
N82-3	3	N82-3-37	Serrated leaf	18:8,430,672 or	1063.5	0	0018s07730	No functional annotation for this gene
		
				15:1,812,282	1059.9	0	0015s02530	Myosin VII
		
		N82-3-66	Shriveled leaf	7:3,447,049	311.5	9.1e-83	0007s05350	Histone binding protein RBBP4

		N82-5-3	Lanceolated leaf	14:11,531,333	450.3	1.6e-124		
		
N82-5	4	N82-5-20	Necrotic leaf	14:2,480,856	302.4	9.4e-81		
		
		N82-5-26	Bonsai plant	n.d.*				
		
		N82-5-28	Bonsai plant	607:2,304	448.5	3.7e-124	0607s00200	No functional annotation for this gene

N82-7	1	N82-7-1	Shriveled leaf	11:13,627,432	565.7	1.7e-159	0011s10950	Protein of unknown function (DUF_B2219)

		N82-11-1	Crippled growth *in vitro*, saw toothed leaf	16:3,641,919	280.8	1.5e-73	0016s05700	Zinc ion binding; nucleic acid binding
		
N82-11	3	N82-11-4	Pale green leaf	n.d.*				
		
		N82-11-5	Pale green leaf	16:13,607,077	426.9	1.4e-117	0016s14360	EF-P Elongation factor

		N82-14-2	Lanceolated leaf	4:23,008,963 or	780.3	0	0004s24320	Protein of unknown function (DUF1218)
		
				17:944,412	1115.8	0	0017s01360	Apoptotic ATPase
		
		N82-14-3	Shriveled leaf	14:6,540,903	904.8	0	0014s08850	Glycosyl hydrolases family 18; Pt-CHI3.5
		
		N82-14-4	Weakly serrated leaf	11:1,379,364 or	535.1	1.2e-150		
		
N82-14	7			3:13,395,264	1079.7	0	0003s12970	No functional annotation for this gene
		
		N82-14-5	Shriveled leaf	10:14,732,583	892.2	0	0010s15550	No functional annotation for this gene
		
		N82-14-6	Weakly shriveled leaf	2:13,782,413	298.8	1.1e-79		
		
		N82-14-9	Shriveled leaf	14:10,351,781	1090.5	0	0014s14140	Protein of unknown function (DUF3754)
		
		N82-14-10	Undulating leaf	17:1,256,405	1204.1	0		

		N82-15-6	Bushy plant	4:9,615,348	1577.4	0	0004s11010	Pentatricopeptide repeat-containing protein
		
N82-15	2	N82-15-10	Lanceolate, serrated	17:2,279,602 or	226.7	5.9e-58	0017s03150	No functional annotation for this locus
		
			leaf	14:14,660,942	226.7	5.2e-58		

N92-1	2	N92-1-1	Variegated leaf	10:15,124,072	147.4	6.0e-34	0010s16070	Stress responsive A/B Barrel Domain
		
		N92-1-6	Variegated leaf	10:15,114,170	206.9	1.2e-51		

N95-1	1	N95-1-1	Variegated leaf	n.d.*				

N95-3	1	N95-3-1	Variegated leaf	n.d.*				

Genomic insertion locus (scaffold and position) with score, e-value and, if applicable, annotated transcript.

In BLAST-analyses where more than one hit was given, either the one with lower e-value or when similar both hits are shown.

*n.d. = not determined.

So far, a new ATDs genomic position could be successfully determined for 24 out of the 29 different putative variants. Sequences for those were blasted against the publicly available genome sequence of *P. trichocarpa *v2.0 (Phytozome v7.0; http://www.phytozome.net/poplar). Resulting e-values ranged from e^-25 ^down to zero. Possible transcripts against *P. trichocarpa *could be annotated for 17 variants. For six lines, putative proteins were of unknown function or no functional annotation was possible (Table 4).

### Suitability of the proof-of-concept approach for large scale transposon tagging in poplar

**Figure 4:**
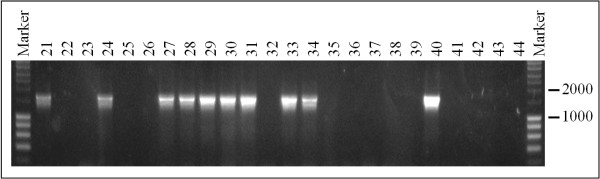
**PCR analysis using the 16/37 primer pair (see Material and Methods) of randomly selected greenhouse-grown plants from different heat shocked double transgenic HSP::*TRANSPOSASE*/ATDs aspen lines**. Following successful excision of ATDs a fragment of 1800 bp in size was obtained. M = Marker (Smart-Ladder; Eurogentec).

Randomly selected 300 greenhouse-grown plants without any obvious phenotypic alterations from 16 different double transgenic HSP::*TRANSPOSASE*/ATDs aspen were PCR-screened for ATDs excision by amplifying a 1,800 bp long region spanning from the *npt-II *to the *rolC *gene using the 16/37 primer pair (Figure 4). The number of tested plants per line varied from 10 to 26. Only in three lines (N92-3, N95-4, N95-5), no ATDs excision could be detected, and in each of N95-1 and N95-2, only two plants were detected (Table 5). Overall, just under one-third of the 300 plants analyzed revealed ATDs excision.

**Table 5:** Heat-shocked and regenerated plants from different HSP::*TRANSPOSASE*/ATDs double transgenic aspen lines without any phenotypic alterations (anonymous approach) grown in the greenhouse were randomly selected and tested for ATDs transposition with the primer pair 16/37.

Transgenic line	Tested plants	PCR-positive (16/37) (%)	TAIL-PCR and positive BLAST hits	Transcript annotation
N82-2	22	3	0	---

**N82-5**	**26**	**11**	**5**	**3**

N82-7	24	4	0	---

N82-11	20	4	1	0

N82-14	13	4	2	0

**N82-15**	**25**	**14**	**8**	**6**

**N92-1**	**19**	**16**	**6**	**4**

N92-2	19	10	0	---

N92-3	20	0	0	---

N92-4	10	6	1	0

N95-1	23	2	0	---

N95-2	19	2	0	---

N95-3	19	9	1	1

N95-4	10	0	0	---

N95-5	10	0	0	---

N95-6	21	10	2	1

SUM (%)	300	95 (32%)	26 (8.7%)	15 (5.0%)

Positive candidates were subjected to TAIL-PCR and sequencing to determine the new ATDs genomic insertion locus. Obtained sequences were blasted against the publicly available genome sequence of *P. trichocarpa *v2.0 (Phytozome v7.0; http://www.phytozome.net/poplar). Numbers of positive BLAST hits and, if applicable, of transcript annotations are given.

In bold the three lines with highest numbers of positive ATDs transposition and BLAST hits.

In order to determine new insertion loci, TAIL-PCR and sequencing was performed in plants that tested positive for ATDs insertion. Resulting sequences were blasted against the publicly available genome sequence of *P. trichocarpa *v2.0 (Phytozome v7.0; http://www.phytozome.net/poplar). In 26 plants (8.7%) from eight lines, TAIL-PCR was successfully conducted and positive BLAST hits were obtained. ATDs flanking genomic sequences could be positively annotated to putative *P. trichocarpa *transcripts for 15 plants (5.0%) belonging to six lines (N82-5, -14, -15, N92-1, N95-3, -6) (Table 5). Out of these, individuals from lines N82-5, N82-15, and N92-1 alone (bold in Table 5) accounted for 13 transcript annotations. A summary of BLAST- and annotation results of the new ATDs flanking sequences is given in Table 6. E-values of hits ranged from e^-17 ^down to zero with exception of three high e-values in N82-5#82, N82-5#213, and N82-15 #4.

**Table 6:** Annotation results of new ATDs flanking sequences in heat-shocked plants from different HSP::*TRANSPOSASE*/ATDs double transgenic aspen lines without any phenotypic alterations ("blind" approach) grown in the greenhouse.

Transgenic line	Number of plants	Plant affiliation	Genomic insertion locus (scaffold:position)*	Score	E-value	Transcript (POPTR_)	Functional annotation
		# 82	16:6,171,964	41.0	5.7e-2		
		
		# 147	8:7,052,973	248.3	1.1e-64		
		
N82-5	5	# 210	9:11,936,320	114.9	1.1e-23	0009s15430	Carotiniod oxygenase
		
		# 211	607:7,883	160.0	3.2e-37	0607s00230	No functional annotations for this locus
		
		# 213	7:1,458,117	42.8	3.52e-3	0007s02460	Ubiquitin-like protein

N82-11	1	# 224	15:11,529,160	1103.1	0		

N82-14	2	# 6	5:23,009,323	482.8	7.9e-135		
		
		# 76	16:13,632,466	452.1	9.8e-126		

		# 4	2:10,051,122	42.8	1.6e-2		
		
		# 73	12:3,471,598	488.2	2.1e-136	0012s04240	ABC-2 type transporter
		
		# 28	19:12,716,418	223.1	1.3e-56	0019s11440	Protein of unknown function (DUF803)
		
		# 110	4:21,317,871	1402.5	0	0004s22400	UDP-glucuronosyl and UDP-glucosyl transferase
		
N82-15	8	# 131	7:13,340,488	389.0	8.8e-107	0007s13230	Dolichyl pyrophosphate phosphatase and related acid phosphatases
		
		# 137	2:10,620,751 and	729.8	0		
		
			2:10,621,198	268.2	1.8e-70	0002s14350	PHD-finger
		
		# 138	20:865,030	91.5	2.4e-17		
		
		# 140	2:21,231,185	524.3	2.1e-147	0002s23690	Cysteine protease inhibitor activity

		# 29	4:1,137,737	771.3	0	0004s01860	Cytochrome P450 CYP2 subfamily
		
		# 85	10:16,307,759	787.6	0	0010s17860	Phosphoglycerate kinase [EC:2.7.2.3 ]
		
N92-1	6	# 151	17:141,236	194.2	2.8e-48	0017s00430	Serine/threonine protein kinase
		
		# 155	8:6,405,007	403.4	5.0e-111		
		
		# 242	10:13,783,139	987.7	0	0010s14080	Diaphanous homolog 2
		
		# 244	4:17,605,886	735.3	0		

N92-4	1	# 93	10:13,739,252	336.7	6.,3e-91		

N95-3	1	# 179	9:9,182,685	253.8	4.2e-66	0009s10820	Apoptosis-promoting RNA-binding protein TIA-1/TIAR

N95-6	2	# 176	1:42,827,910	740.7	0		
		
		# 190	1:2,422,398	459.3	8.0e-128	0001s03080	Type II intron maturase

Genomic insertion locus (scaffold and position) with score, e-value and, if applicable, annotated transcript.

In BLAST-analyses with more than one hit the one with lower e-value is shown.

## Discussion

Different mutagenesis approaches based on heterologous (transferred) transposon element systems have been successfully applied in many plant species. Most prominently, the two element maize *Ac*/Ds system has been successfully used to generate insertional mutants in Arabidopsis, rice or barley [12,27-31]). In order to establish a similar transposon tagging system for trees, Fladung and Ahuja [24] transferred the autonomous *Ac *element to aspen-*Populus *and for the first time confirmed that *Ac *is functionally active in this tree species. Molecular evidence for *Ac *excision and re-integration into the genome was later provided by Kumar and Fladung [23]. Further, these authors showed that the majority of *Ac *genomic re-integration sites were found within or near coding regions. More recently, Fladung [25] analyzed in detail the genomic positions of *Ac *re-integrations by blasting *Ac*-flanking aspen sequences against the publicly available genome sequence of *P. trichocarpa *v2.0 (Phytozome v7.0; http://www.phytozome.net/poplar). The majority of re-integrations were found scattered over many unlinked sites on different scaffolds confirming that in poplar *Ac *is able to cross chromosome boundaries. These latest results confirmed the feasibility of the approach first suggested by Kumar and Fladung [23] to use the *Ac*/Ds transposon tagging system for functional genomics studies in forest tree species, and in particular, for an efficient induction of mutants.

In this study, we took advantage of the already available "Activation Tagging Ds" system (ATDs) developed by Suzuki et al [26] that contains outwards directed 35S promoters at both ends. For our study, this ATDs system was combined with the phenotypic selectable marker gene *rolC *[23,32], which was cloned outside of the ATDs element so that it is active when ATDs is not excised. This gene construct was transformed into two already transgenic *TRANSPOSASE*-expressing aspen-*Populus *lines. A gain-of-function rather than a loss-of-function strategy was used as this approach does not disrupt gene expression, avoids issues of gene redundancy and allows screening to occur in a primary generation. In earlier work, an "Activation tagging" approach has been recommended as particularly practicable for application in long-living trees [13,22].

To date, successful T-DNA-based activation tagging mutagenesis in trees has been reported only for poplar [14,15] and *GA2-OXIDASE*, a gibberellin catabolism gene, was the first tree gene that was isolated from a poplar T-DNA insertion population comprising 627 individuals [22]. In the following years, other T-DNA activation tagging poplar populations were produced and screened for developmental abnormalities including alterations in leaf and stem structure as well as overall stature by Harrison et al. [21] The mutant frequency reported for the largest activation tagging poplar population (with 1,800 independent transgenic lines) was about 2.4%. In contrast, in our study, a total of 12,083 individuals were produced and screened, but our visible mutant frequency (containing also leaf and stem phenotypic alterations) was only 0.24%. However, in an additional "blind" approach (without any previous phenotyping), we determined a frequency of 32% of ATDs transpositions in randomly selected heat shocked plants. Thus, by considering only positive ATDs-tested (transposed) individuals, the mutant frequency could be raised to approximately 1%. At present, we are working on a further increase of the mutant frequency by using a positive reporter gene system combined with the ATDs system. This system only allows shoots to regenerate when the reporter gene is not active any more and thus ATDs is excised.

Thus, critically to our heat shock-based *TRANSPOSASE*-induction strategy, the heat shock regime itself seems to influence ATDs excision rate. This is consistent with observations made in a carefully performed study on flowering response following heat shock induction of the *FT *gene controlled also by the soybean Gmhsp17.5-E heat shock promoter, in which both the size of the treated plants as well as the temperature regime influenced success of flower induction [33]. Daily heat treatments (1-2 hours at 37°C) over a period of three weeks or heat treatments of shorter durations but with increased inductive temperature (from 37°C to 40°C) were reported to be successful for efficient flower induction in greenhouse grown plants taller than 30 cm [33]. In a previous study on the induction of a FLP/*FRT *recombination system, the soybean heat shock promoter was induced after incubation of *in vitro *grown transgenic poplar plants and regenerative calli at 42°C for 3 hours [34]. Transposase induction following heat treatment of *in vitro *grown individuals from double transgenic lines was also confirmed by RT-PCR (data not shown).

Possible explanations for the overall relatively low frequency of ATDs transposition could be silencing effects due to double insertion of the ATDs element or chromosomal position of the original (donor) ATDs locus. Early evidence for a relationship between T-DNA copy number and repeat formation as well as promoter methylation in poplar has been provided by Kumar and Fladung [35]. However, among the 23 different double transgenic lines carrying one to four copies of ATDs, no notable correlation was found between copy number and mutant frequency.

Alternatively, in ten (N82-3, -4, -5, -7, -8, -10, -11, N92-3, N95-2, -3) out of 23 primary double transgenic, non-ATDs transposed lines, annotations of the ATDs donor locus flanking genomic sequences revealed insertion into or nearby genes. These ten lines, which themselves can be considered as T-DNA tagged variants, yielded only twelve ATDs-tagged variants. On the contrary, analysis of genomic sequences flanking ATDs donor loci in the two lines with the highest number of phenotypically tagged lines (N82-2 with 5 and N82-14 with 7) revealed no transcript annotation. A similar trend was observed in our anonymous approach. Here, randomly selected heat-shocked plants were first PCR-screened for successful ATDs excision, and, in a second step, ATDs excision-positive plants were analyzed for genomic localization of new ATDs insertion sites. Out of 128 tested plants from six of the above mentioned ten lines with annotations, 30 positive ATDs excisions (23.4%) and 7 BLAST hits (5.5%) were detected. However, three lines without any positive annotation of the ATDs donor locus flanking genomic sequences (N82-14, -15, N92-1) revealed 34 positive ATDs excisions (59.6%) and 16 BLAST hits (20.2%) in 57 tested plants.

The variations in phenotype in some of the ATDs-tagged mutants might be similar to those observed by Harrison [36] explaining partial silencing of the *shriveled leaf *mutant due to methylation effects. A positive correlation between 35S enhancer element methylation and low frequency of T-DNA-based activation tagging was reported by Chalfun-Junior et al. [37] Further, an early report describes the influence of endogenous and environmental factors on 35S promoter methylation [38]. Because ATDs is carrying both the four repeats of enhancer elements as well two 35S promoters, variations of mutant phenotypes are possible. Further, to confirm that the variants obtained are truly transposon-tagged and possibly also to explain observed phenotype variations, we already have initiated heat-shock treatments of some variants to restore the wildtype phenotype. Further, semi-quantitative PCR analyses are underway to confirm the activation of the transcripts in the variant lines.

Tagging approaches based on T-DNA insertion are effective only for plant species (like Arabidopsis and poplar) that can be easily transformed and for which high frequencies of tagged lines can be obtained [28]. One possible advantage of T-DNA based activation tagging could be that even T-DNA insertion sites are not randomly distributed in the genome but do show some insertion site preferences to the 5'UTR of a gene coding region [39]. For transposable elements, however, new insertion sites were found scattered throughout the genome at many unlinked sites [28,31,40]. But similar to results obtained for poplar [25], also for Arabidopsis a preferential transposon insertion around transposon donor sites was found by Raina et al. [41] However, in many of the heat shocked ATDs-excision positive tested plants analyzed in this study, the scaffolds revealing the new ATDs insertion loci are unlinked to those harbouring the original donor locus.

## Conclusions

The fact that a transposon is able to jump to other chromosomes, thus passing chromosomal boundaries, leads to the convenient situation that only a few primary transposon transgenic lines are required for the establishment of large transposon tagging populations in order to tag at least theoretically every gene in a tree genome. This would be difficult to achieve through T-DNA tagging as plant transformation is time consuming and, therefore, the genome can't easily be saturated with T-DNA tags.

For both T-DNA and transposon activation tagging, the strategy followed so far was to first phenotype an existing tagging population and then to determine new genomic insertion loci of a tag. Based on our results presented here, we propose a novel strategy of activation tagging that is supported by the demonstrated power of the ATDs transposon approach and the simplicity to induce ATDs transposition *in vitro *at least in some lines. The ATDs-based strategy allows first the production of a very high number of independent ATDs-transposed plants that can be screened for new ATDs flanking genomic loci. The sequences obtained in this way can then be subjected to BLAST analyses, and finally based on this *in silico *research, variants of specific interest can be selected, transferred to and investigated in the greenhouse.

## Methods

### Plasmids

**Figure 5:**
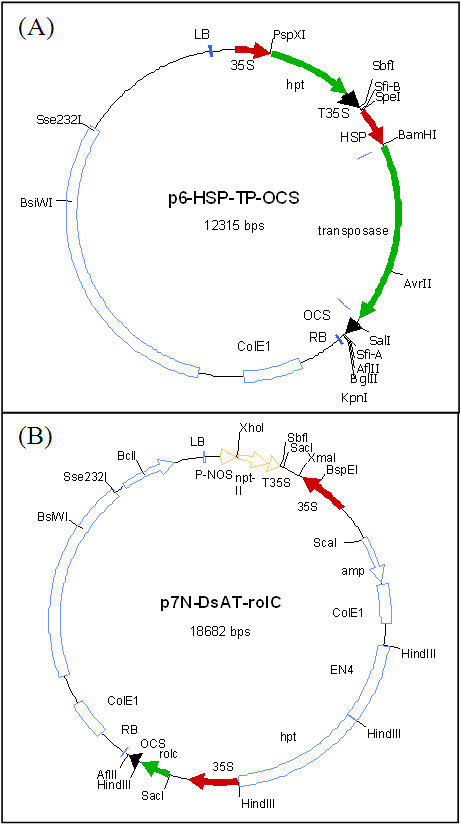
**Schematic representation of the binary vectors p6-HSP-TP-OCS (A) and p7N-ATDs-*rolC *(B) used for the transformation experiments**. (A) Structure of the plasmid containing the *Ac *transposase and heat shock promoter (HSP) Gmhsp17.5-E from soybean [42], and *hpt *as plant selectable marker. (B) Structure of the activation construct (ATDs) [26]. The ATDs contains *hpt *as transposition selection marker. The *rolC *gene is outside of ATDs and the CaMV 35S promoter on the left site of the ATDs keeps this gene active under non-excised stage. The *nptII *gene with the nopaline synthase promoter (nos) is used as transformation marker. Relevant restriction enzyme cutting sites are introduced into the maps. (P-NOS = Nopaline synthase promoter; HSP = GmHSP 17.5-E promoter; nptII = *neomycin phosphotransferase *gene; hpt = *hygromycin phosphotransferase gene*; 35S = CaMV35S promoter; T35S = CaMV35S terminator; OCS = *Octopine synthase *gene terminator; EN4 = four tandem repeats of enhancer fragments; rolC = *rolC *gene from Agrobacterium rhizogenes; LB and RB = Left- and right border sequences).

Two constructs formed the basis for our experiments. The first construct, p6-HSP-TP-OCS, carries the *TRANSPOSASE *gene from the *Ac *element from maize under control of the soybean heat shock promoter (HSP) Gmhsp17.5-E [42] and is similar to the heat-shock inducible transposase system described by Czarnecka et al [43] (Figure 5a). As plant selectable marker this construct carries the hygromycin resistance gene (*hpt*) under control of the cauliflower mosaic virus (CaMV) 35S promoter.

The second construct, p7N-ATDs-*rolC *(Figure 5b), comprises the "Activation Tagging Ds" system (ATDs) kindly provided by Y. Suzuki, University of Tokyo, Tokyo Japan [26] and the *rolC *gene from *Agrobacterium rhizogenes *which functions as phenotypic selectable marker ([23,24] for transposition of the ATDs (Figure 5b). The ATDs is flanked by terminal inverted repeats and contains two CaMV 35S promoters facing outward as well as four tandem repeats of enhancer fragments (En) of the 35S promoter that work for promoter-type and enhancer-type gene activation, respectively. The *rolC *marker gene is located outside of the ATDs element, and following excision of ATDs, *rolC *becomes promoterless und thus inactive. For selection of transgenic plants the ATDs construct carries the *nptII *selectable marker gene.

Both gene constructs were transferred into the *Agrobacterium tumefaciens *strain GV3101::pMP90RK [44].

### Transformation of aspen with the *TRANSPOSASE *gene and selection of two transgenic lines for super-transformation

The aspen hybrid clone "Esch5" (*P. tremula *L. *× P. tremuloides *Michx.) was first transformed with p6-HSP-TP-OCS carrying the *TRANSPOSASE *gene using a *Agrobacterium*-mediated leaf disc co-cultivation method as described by Fladung et al [45] and Hoenicka et al. [46] For selection of transgenic plants, the regeneration media contained hygromycin (20 mg/L) and Cefotaxime (500 mg/L). In total, seven independent transgenic lines tested positive for presence of a HSP-*TRANSPOSASE *fragment in PCR experiments (data not shown). Further, a RT-PCR approach was followed to assess transposase transcription in the seven HSP-*TRANSPOSASE *transgenic lines following a 24 h culture at 37°C under continuous light. Induction of transposase transcription was observed in all investigated HSP-*TRANSPOSASE *transgenic lines, thus, this treatment was sufficient to induce the transposase without inflicting noticeable stress on the plants (data not shown). In order to show that transposase transcription did not occur in non-heat-shock-treated plants (due to theoretically possible leakage of the HSP promoter), non-induced leaves were included in RT-PCR experiments, and no such transcription was detected (data not shown). Finally, four weeks after heat-shock treatments RNA was again isolated from leaves of two HSP-*TRANSPOSASE *transgenic lines and RT-PCR experiments were performed, confirming that four weeks after treatment transposase transcripts are no longer detectable (data not shown). Based on these results, two independent transgenic lines, N66-2 and N66-5, were chosen for super-transformation with ATDs.

### Super-transformation of N66-2 and N66-5 with p7N-ATDs-*rolC*

For the two independent *TRANSPOSASE*-transgenic aspen lines N66-2 and N66-5, an *Agrobacterium*-mediated super-transformation with the p7N-ATDs-*rolC *construct (carrying the ATDs element) was performed. In total, 13 and twelve independent double transgenic lines were obtained for N66-2 and N66-5, respectively, and analysed in PCR experiments for presence of this second gene construct (data not shown). Complete integration of this gene construct was tested using primer pairs that cover the region from the 35S-promoter of the ATDs element to flanking genes (either *rolC *[at right border of T-DNA] or *nptII *[at left border of T-DNA], see Figure 5b). For 23 transgenic lines complete integration of the ATDs construct was confirmed (twelve lines for N66-2 and eleven lines for N66-5) (data not shown). Affiliations of the p7N-ATDs-*rolC*-transgenic lines for N66-2 are: N82-2, N82-3, N82-4, N82-5, N82-7, N82-8, N82-10, N82-11, N82-12, N82-13, N82-14, N82-15, and for N66-5: N92-1, N92-2, N92-3, N92-4, N92-6, N95-1, N95-2, N95-3, N95-4, N95-5, N95-6. These lines were considered for the activation tagging experiments described below.

### DNA extraction, PCR confirming ATDs excision, and Southern blot analyses

Genomic DNA was isolated from transgenic aspen lines using the CTAB method described by Dumolin-Lapègue et al [47], and total RNA was purified according to Logemann et al [48]. DNase-treatment of purified RNA was done with RNAfree DNAse (Cat. Nr. M6101, Promega, Mannheim, Germany) followed by transposase transcription using the Access RT-PCR System (Cat. Nr. A1250, Promega, Mannheim, Germany).

Excision of the ATDs fragment after heat shock treatment was verified by PCR using the primer pair *nptII *(forward [F]; internal primer number #16, 5'-ATG GAT TGC ACG CAG GTT CTC-3') and *rolC *(reverse [R]; internal primer number #37; 5'-AAC TCA CCA GGT TCG AAC CTA-3'). Following successful excision of ATDs, a fragment of 1800 bp in size was obtained.

Copy numbers of the *TRANSPOSASE *and ATDs constructs were determined by Southern blot analyses using DIG-labelled DNA-probes specific for *TRANSPOSASE *and *hpt *(for N66-2 and N66-5), and *nptII *and *rolC *(for eleven N82er-, five N92er-, and six N95er transgenic lines).

For Southern blot analyses, 20 μg of genomic DNA was cleaved with *Bam*HI (N66-2, N66-5), and *Sca*I or *Sac*I (for N82er-, N92er-, and N95er transgenic lines). Restricted DNA samples were separated on 1.3 or 1.5% agarose gels in TAE buffer, blotted on nylon membranes and hybridised with DIG-labelled DNA probes as described by Fladung et al [45]. DIG-labelling of all DNA-probes was done in a PCR reaction according to Fladung and Ahuja [24] using the following primer pairs: *TRANSPOSASE *(F; #1: 5'-AAT AAG TCA TAC ATG TGT GTC ACC-3'/R; #2: 5'-TAC AAT TTT CTA ATG ACC CTA ACA-3'); *hpt *(F; #337: 5'-AAA GCC TGA ACT CAC CGC GA-3'/R; #338:5'-TCG GTT TCC ACT ATC GGC GA-3') *nptII *(F; #16: 5'-ATG GAT TGC ACG CAG GTT CTC-3'/R; #17: 5'-AAG GCG ATA GAA GGC GAT GCG-3') *rolC *(F; #6: 5'-GGC TGA AGA CGA CCT GTG TTC TCT-3'/R; #37: 5'-AAC TCA CCA GGT TCG AAC CTA-3').

### TAIL-PCR for determination of genomic integration sites

TAIL-PCR was performed as described by Liu et al [49] with the following modifications. Annealing temperatures during PCR reactions in TAIL1, TAIL2 and TAIL3 were adapted to the requirements of the specific primer used. For TAIL1, 200 ng of genomic DNA was added to the reaction mix, and TAIL1 products were diluted 1:50 with water for TAIL2, and 1 μl of TAIL2 was directly taken for TAIL3. Taq-polymerase and PCR buffer from the Expand Long Range dNTPack (Roche, Germany) were used for PCR reaction instead of standard Taq.

All three arbitrary degenerate (AD) primers were tested in combination with three specific primers designed for the left and right borders of p6-HSP-TP-OCS (carrying the *TRANSPOSASE *gene) and p7N-ATDs-*rolC *(carrying ATDs). The 64-fold degenerate AD-primer (5'-NTC GAS TWT SGW GTT-3') was most successful in generating fragments in the three subsequent TAIL-PCR rounds in combination with the construct specific primers 1, 2, and 3 (see below). In cases where no PCR products were obtained with the 64-fold degenerate AD primer, the 128-fold (5'-NGT CGA SWG ANA WGA A-3') or even the 256-fold (5'-WGT GNA GWA NCA NAG A-3') degenerate AD-primer was used for the PCR reactions.

For TAIL-PCR amplification of genomic left and right border flanking regions of the p6-HSP-TP-OCS construct (transgenic plant lines N66-2, N66-5), the following specific primers were used in combination with the AD primers: left border: primer 1: 5'-TGG GAT TGT GCG TCA TCC CT-3'; primer 2: 5'-ATC CGA GGA GGT TTC CCG-3'; primer 3: 5'-GAC GGA TCG TAA TTT GTC GTT-3'; right border: primer 1: 5'-AGG ATT ATG ATC AAG TAG AGT C-3'; primer 2: 5'-AAG ATT GGG TAG CAG CAT CTA-3'; primer 3: 5'-TCA GAT CCT TAC CGC CGG TTT-3'.

For amplification of the original genomic insertion locus of the p7N-ATDs-*rolC *construct the following primers were used in combination with the AD primers: left border: primer 1: 5'-AAG GCG ATA GAA GGC GAT GCG-3'; primer 2: 5'-TTC AAC GTT GCG GTT CTG TCA-3'; primer 3: 5'-GAC GGA TCG TAA TTT GTC GTT-3'; right border: primer 1: 5'-TTA TAC GAT AAC GGT CGG TAC-3'; primer -2: 5'-ACT GCC CGA CGA TGA TGC TCT-3'; primer -3: 5'-TCA GAT CCT TAC CGC CGG TTT-3'.

Following heat shock, the new genomic positions of the ATDs element were determined by using the following primers in combination with the AD primers: left site of ATDs: primer 1: 5'-AGT CCA AAT CGG ATC TGT AAG-3'; primer 2: 5'-ACC GAA CAA AAA TAC CGG TTC-3'; primer 3: 5'-CGA TTA CCG TAT TTA TCC CGT-3'; right site of ATDs: primer 1: 5'-TGC AGT CAT CCC GAA TTA GAA-3'; primer 2: 5'-CGT TTC CGT TTA CCG TTT TGT-3'; primer 3: 5'-TTA TAC GAT AAC GGT CGG TAC-3'.

Fragments obtained were eluted from agarose (Qiagen, Duesseldorf, Germany) and sequenced (StarSeq, Mainz, Germany). All sequences obtained were blasted against the publicly available genome sequence of *P. trichocarpa *v2.0 (Phytozome v7.0; http://www.phytozome.net/poplar).

Successful BLAST-results were used to position the T-DNA on the physical map of *P. trichocarpa*. These positions could be assigned to the *Populus*-aspen genome because of the high collinearity between the *P. trichocarpa *and *P. tremula/P.tremuloides *genomes [50].

All aspen-specific sequences obtained in this study were integrated into GabiPD database (http://www.gabipd.org) and submitted to GenBank ([GenBank:JM973488] to [GenBank:JM973566]).

### Heat shock treatments to induce ATDs transposition

Four different heat shock experiments were conducted with 23 independent double transgenic HSP::*TRANSPOSASE*/ATDs aspen lines (Table 3). To activate the ATDs transposition system, transgenic regenerative callus cultures, including regenerated poplar shoots, were incubated at various temperature regimes as shown in Table 3. Treatment conditions were either 16 or 24 hours at 42°C (experiments 1, 2, and 4) or 8 hours at 42°C applied over three subsequent days (experiment 3).

One to twenty four hours after the heat shock treatment, regenerative callus, leaves and stems were crushed into small pieces by using a Waring blender (pieces as small as possible but without destroying individual cells). The resulting "cell-pulp" was transferred to fresh regeneration medium (Figure 2) and cultivated for up to 5 months at 25°C under continuous light in a growth chamber. Regenerated shoots were cut and transferred to WPM medium without rooting hormones. After 4 to 8 weeks, rooted plants were phenotyped in tissue culture for the first time. Rooted plants were transferred into soil and phenotyped again after three to six months of growth in the greenhouse.

### Phenotyping of *in vitro*- and greenhouse grown plants

Rooted plants *in vitro *were screened for growth deficiency and chlorophyll abnormalities. In the greenhouse, up to six-months-old plants were screened for phenotypic variations as well as for leaf form and shape alterations.

For the "blind" approach, 300 greenhouse-grown plants without any obvious phenotypic alterations were randomly selected from 16 different double transgenic HSP::*TRANSPOSASE*/ATDs aspen lines and PCR-screened for ATDs transposition using the 16/37 primer pair as described above. Plants that tested positive were further screened by TAIL-PCR for new genomic location of the ATDs as described above with the exception that a standard Taq-polymerase (DNA Cloning Service, Hamburg, Germany) was used instead of the Long-Range Taq. Fragments obtained were sequenced (StarSeq, Mainz, Germany), and the sequences were blasted against the publicly available genome sequence of *P. trichocarpa *(assembly v2.0; Phytozome v7.0; http://www.phytozome.net/poplar).

## Authors' contributions

This study was conceptualized planned by MF. All experimental work was conducted by OP, supervised by MF. The paper was written by MF. All authors have read and approved the final manuscript.
